# *Plasmodium vivax* cerebral malaria with pancytopenia in the peruvian amazon: case report

**DOI:** 10.17843/rpmesp.2022.392.10739

**Published:** 2022-06-30

**Authors:** Marco Paredes-Obando, Alfrando Moreno, Eduardo Panduro-García, André Ferreyra, Diego Chuquipiondo-Galdos, Jhosephi J. Vásquez Ascate, Jorge Sibina-Vela, Edgar A. Ramírez-García, Juan C. Celis-Salinas, Martín Casapía-Morales

**Affiliations:** ¹ Facultad de Medicina Humana de la Universidad Nacional de la Amazonía Peruana, Iquitos, Peru. Universidad Nacional de la Amazonía Peruana Facultad de Medicina Humana Universidad Nacional de la Amazonía Peruana Iquitos Peru; ² Hospital Regional de Loreto, Iquitos, Peru. Hospital Regional de Loreto Iquitos Perú

**Keywords:** Cerebral Malaria, *Plasmodium vivax*, Seizures, Pancytopenia

## Abstract

*Plasmodium vivax* causes 81% of all malaria cases and is the most common species in the Peruvian Amazon. We present the case of a male patient with cerebral malaria caused by *Plasmodium vivax*, who had general malaise and fever, and then presented seizures more than twice a day with loss of consciousness and motor functional limitation. *Plasmodium vivax* trophozoites were detected by thick blood smear, besides, we also observed low counts of all three blood cell types. Treatment began with artesunate and clindamycin for five days, then one unit of packed red blood cells was transfused; treatment continued with primaquine for seven days. The patient showed clinical improvement with neurological sequelae in one lower limb.

## INTRODUCTION

Cerebral malaria (CM) is a complication of severe malaria, characterized by diffuse encephalopathy associated with coma and convulsions, most of the reported cases are due to *Plasmodium falciparum* infection in children ^1^. Generally, *Plasmodium vivax* only causes uncomplicated malaria; however, some cases of complicated malaria have been reported, although cerebral involvement is rare ^2^. Cases of malaria by *Plasmodium vivax* represent 81% of all cases diagnosed in Peru, with an incidence of 42.68 cases per 100,000 inhabitants; and are more frequent in men (54%) ^3^ ^,^ ^4^. A study conducted in the tropical zone of Piura in Peru during 2008 reported 0.4% of critical patients of which only 11% (three individuals) had CM ^5^, therefore this type of malaria should be considered as a differential diagnosis of encephalitis in tropical areas. Laboratory findings are characterized by leukocytosis. ^6^. This report describes a case of an adult patient with *P. vivax* CM associated with pancytopenia.

## CASE REPORT

A 30-year-old male patient from the town of Intuto, located 172 km from Iquitos, in the Peruvian Amazon. The patient had history of untreated type 2 diabetes *mellitus*. He was admitted to the emergency department of the Regional Hospital of Loreto for convulsions, loss of consciousness and functional limitation, associated with chills, headache, feverish feeling and poor general condition. On clinical examination he presented the following vital signs: BP: 90/60 mmHg; HR: 67/min; RR: 20/min: T°: 36.5 °C; oxygen saturation: 96%; weight: 60 kg; BMI: 19.6 m^2^. Besides, he presented pallor ++/+++, prostration, general malaise, cold extremities, sensory disturbance, myalgia and arthralgia.

The illness lasted 14 days and began with seizures of 1 to 2 min with a frequency of 5 to 6 times a day. Two days before admission, the patient experienced loss of consciousness and motor functional limitation, so he was transferred to the nearest health center. In this health center he underwent thick and thin blood smear tests, which showed *Plasmodium vivax* trophozoites and therefore, treatment was initiated. However, the physician decided to refer him to a higher complexity health center, therefore, the patient was admitted to the emergency department of the Loreto Regional Hospital where he was hospitalized

From admission, the patient was treated with artesunate 14.4 mg, which was repeated at 12 and 24 h; all doses were diluted with 5% dextrose in a volume of 5 to 10 cc in a 5 min bolus. Then, treatment continued with artesunate 24 mg PO until the fifth day. Intravenous clindamycin 600 mg diluted in 50 mL of 0.9% sodium chloride was administered in infusion for 20 to 30 min every 12 h for 5 days simultaneously with the start of intravenous artesunate. Subsequently, one unit of packed red blood cells was transfused, after which, treatment continued with primaquine for 7 days.

*Plasmodium vivax *wasn’t found during the thick blood smear test performed after completing the treatment. Laboratory tests showed low counts of all three blood cell types; the peripheral blood smear showed typical granulocytes ++ and atypical lymphocytes +. The erythrocytic series showed microcytosis with anisocytosis and basophilic stippling (Table 1). Abdominal ultrasound showed splenomegaly and bilateral pleural effusion. The patient underwent lumbar puncture and the cerebrospinal fluid was analyzed (Table 2). The patient showed clinical improvement with neurological sequelae of pure motor monoparesis in the left lower limb.

**Table 1:** Laboratory tests on whole blood

Type of test	Day 1 (31-08-21)	Day 2 (01-09-21)	Day 3 (02-09-21)
Leucocytes	NP	2.39 x 10³/uL	3.37 x 10³/uL
Platelets	NP	153 x 10³/uL	339 x 10³/uL
Erythrocytes	NP	2.22 x 10³/uL	2.19 x 10³/uL
Hemoglobin	NP	6.6 g/dL	6 g/dL
Hematocrit	NP	19.4%	19.4%
Thick drop smear	*Plasmodium vivax* (+++)	Negative	NP
HIV rapid test	NP	Negative	NP

NP: not performed

**Table 2:** Laboratorial tests on cerebrospinal fluid samples

Type of test	Result
Leucocytes	2 leucocytes/mm³
Erythrocytes	10 x 12 x field
Glucose	78.3 mg/dL
Proteins	28.5 mg/dL
Gram stain	No germs observed
Ziehl-Neelsen stain	No AFB observed
Negative stain	Negative

AFB: Acid-fast bacillus

## DISCUSSION

CM is mostly caused by severe *Plasmodium falciparum* infection, with inflammation playing a significant role in the process of neuronal damage. Clinical symptoms are heterogeneous and outcomes range from complete recovery to varied neurological sequelae and sometimes death ^7^ ^,^ ^8^. Typically, CM by *Plasmodium vivax* is attributed to a mixed infection with *Plasmodium falciparum* parasites. Therefore, it is advisable to confirm *Plasmodium vivax* monoinfection with reliable tests such as polymerase chain reaction (PCR); however, the response to specific treatment against *Plasmodium vivax* infection could explain the monoinfection ^1^ ^,^ ^15^. We present a case of malaria by *Plasmodium vivax* detected by conventional methods, which showed neurological symptoms and responded to antimalarial therapy.

The patient had several seizure episodes, followed by confusion and poor response to stimuli, similar to patients with acute infection who develop diffuse encephalopathy, rapid progressive coma and/or seizures without return of consciousness. In some cases, there are also focal neurological signs ^9^. However, seizure is not a criterion for the diagnosis of CM, but of severe malaria. Therefore, since this was the case of a febrile patient from a malaria endemic area, with a sensorimotor disorder without any other possible cause, the diagnosis of CM was considered.

Thirteen children were diagnosed with PCR-confirmed CM by* Plasmodium vivax* in a case series conducted between 2008 and 2010 in India. Eleven of them had multiple seizures, and seven also had signs of first motor neuron involvement, without evidence of hemorrhage or papilledema ^10^. However, a case report from 2016 in the same country, presented the case of a 10-year-old boy with multifocal hemorrhage and severe sensorimotor involvement who, at hospital discharge, presented neurological sequelae such as increased muscle tone and minimal consciousness ^2^.

The anemia and the decrease in platelet count are suggestive of severe malaria; which along with leukopenia indicate low counts of all three blood cell types. There are not many reports of cases in which all three blood cell types are altered with malaria as the sole cause ^11^. Given that this pattern is unusual, a hematologic evaluation was carried out to search for an external cause of the disease, however, the specialist’s report does not suggest any cause other than malaria.

In most reported cases, initial diagnosis was made by direct observation of the parasite with thick blood smear and peripheral blood smear tests, and identification of the species by molecular techniques (PCR) ^1^ ^,^ ^2^ ^,^ ^11^ ^-^ ^13^. 

The response to treatment with artesunate and clindamycin was optimal; it should be noted that artesunate is the treatment of choice for severe malaria ^1^ ^,^ ^2^ ^,^ ^11^ ^-^ ^13^ and that the second drug may vary, which can be clindamycin ^2^, doxycycline ^1^ ^,^ ^13^, lumefantrine ^12^ and even quinine and primaquine without the use of artesunate ^14^. All these schemes have proven to be effective and have shown good results in blood tests after treatment.

A limitation of this report is that it was not possible to use PCR for the diagnosis of CM, due to the limited medical infrastructure in the area.

In this case, *Plasmodium vivax* was identified as the causal agent of CM in the patient. This parasite should be considered as a presumptive diagnosis in cases of neurological involvement in malaria-endemic tropical areas and not think only of *Plasmodium falciparum* as the sole cause of this complication.
