# Supplementary material for: Plasmodium vivax cerebral malaria with pancytopenia in the peruvian amazon: case report
Source: Rev Peru Med Exp Salud Publica. 2022 Jun 30;39(2):241–4. doi: 10.17843/rpmesp.2022.392.10739 (PMC11404674; doi:10.17843/rpmesp.2022.392.10739)
Supplement: Translation

# Translation: *Malaria cerebral con pancitopenia por *italic>plasmodium vivax en la amazonía peruana: reporte de caso

**Authors:** Marco Paredes-Obando, Alfrando Moreno, Eduardo Panduro-García, André Ferreyra, Diego Chuquipiondo-Galdos, Jhosephi J. Vásquez Ascate, Jorge Sibina-Vela, Edgar A. Ramírez-García, Juan C. Celis-Salinas, Martín Casapía-Morales

**Affiliations:** 1 Facultad de Medicina Humana de la Universidad Nacional de la Amazonía Peruana, Iquitos, Perú.; 2 Hospital Regional de Loreto, Iquitos, Perú.

**Keywords:** Malaria Cerebral, *Plasmodium vivax*, Convulsiones, Pancitopenia

## Abstract

*Plasmodium vivax* es la especie más común en la Amazonía peruana y ocasiona el 81% del total de casos de malaria. Presentamos el caso de un paciente adulto varón con malaria cerebral por *Plasmodium vivax*, que inicia con malestar general y fiebre, luego presenta convulsiones más de dos veces al día con pérdida de consciencia y limitación funcional motora. Se le realiza gota gruesa donde se observa trofozoítos de *Plasmodium vivax* y depresión de las tres series sanguíneas. Se inicia tratamiento con artesunato y clindamicina por cinco días, se le transfunde un paquete globular, y continua con primaquina por siete días. El paciente muestra mejoría clínica con secuela neurológica en extremidad inferior izquierda.

## INTRODUCCIÓN

La malaria cerebral (MC) es una complicación de la malaria grave, caracterizada por encefalopatía difusa asociada a coma y convulsiones, la mayoria de los casos reportados son por infección de *Plasmodium falciparum* en niños ^1^. Generalmente, *Plasmodium vivax* solo causa paludismo no complicado; sin embargo, se han reportado algunos casos de malaria complicada, pero es poco usual la afectación cerebral ^2^. Los casos de malaria por *Plasmodium vivax* representan el 81% de todos los casos diagnosticados en el Perú, con una incidencia de 42,68 por cada 100 mil habitantes y mayor frecuencia en hombres (54%) ^3^ ^,^ ^4^. Un estudio realizado en la zona tropical de Piura en Perú, en el 2008, reportó 0,4% de pacientes críticos de los cuales solo 11% (tres individuos) tuvieron MC ^5^, por lo cual este tipo de malaria debería ser considerado como diagnóstico diferencial de encefalitis en los trópicos. Caracterizado laboratorialmente por cursar con leucocitosis ^6^. En el presente reporte se describe un caso de un paciente adulto con MC por *P. vivax* asociado a pancitopenia. 

## REPORTE DE CASO

Paciente varón de 30 años procedente de la ciudad de Intuto, ubicado a 172 km de ciudad de Iquitos, en la Amazonía peruana. Con antecedente de diabetes *mellitus* tipo 2 sin tratamiento. Acude al servicio de emergencia del Hospital Regional de Loreto por convulsiones, pérdida de la conciencia y limitación funcional, acompañado de escalofríos, cefalea, sensación de alza térmica y mal estado general. Al examen clínico tuvó los siguientes signos vitales: PA: 90/60 mmHg; FC: 67/min; FR: 20/min: T°: 36,5 °C; saturación de oxígeno: 96%; peso: 60 kg; IMC: 19,6 m². También presentó palidez ++/+++, postración, malestar general, frialdad de extremidades, trastorno del sensorio, mialgia y artralgia.

El tiempo de enfermedad duró 14 días e inició con convulsiones de 1 a 2 min con una frecuencia de 5 a 6 veces al día. Dos días antes del ingreso presentó pérdida de conciencia y limitación funcional motora, por lo que es trasladado al centro de salud más cercano, donde le realizan un examen de gota gruesa y extendido, con observación de trofozoítos de *Plasmodium vivax* e inicio de tratamiento. Sin embargo, el médico decidió referirlo a un centro de salud de mayor complejidad, por lo que el paciente acude al servicio de emergencia del Hospital Regional de Loreto donde es hospitalizado.

Desde el ingreso el paciente recibe tratamiento con artesunato endovenoso 14,4 mg, que se repitió a las 12 y 24 h, todas las dosis fueron diluidas con dextrosa al 5% en volumen de 5 a 10 cc en bolo de 5 min. Se continuó con artesunato a 24 mg vía oral hasta el quinto día. Simultáneo al inicio de artesunato endovenoso, se administró clindamicina 600 mg diluida en 50 mL de cloruro de sodio al 0,9% en infusión durante 20 a 30 min cada 12 h por 5 días. Posteriormente, se le transfundió un paquete globular, luego de lo cual se continuó con primaquina por 7 días.

Luego de haber recibido el tratamiento, se le realiza nuevamente el examen de gota gruesa sin observación del parásito. En los exámenes de laboratorio se evidencia depresión de las tres series de células sanguíneas con extendido de lámina periférico, granulocitos típicos ++, linfocitos atípicos +. En la serie eritrocitaria se observó microcitosis con anisocitosis y punteado basófilo (Tabla 1). La ecografía abdominal muestra esplenomegalia y derrame pleural bilateral. Se le realiza punción lumbar con análisis de líquido cefalorraquídeo (Tabla 2). El paciente muestra mejoría clínica con secuela neurológica de monoparesia motora pura en inferior izquierda.

**Tabla 1:** Exámenes laboratoriales en sangre total.

Tipo de examen	Día 1 (31-08-21)	Día 2 (01-09-21)	Día 3 (02-09-21)
Leucocitos	NR	2,39 x 10³/uL	3,37 x 10³/uL
Plaquetas	NR	153 x 10³/uL	339 x 10³/uL
Eritrocito	NR	2,22 x 10³/uL	2,19 x 10³/uL
Hemoglobina	NR	6,6 g/dL	6 g/dL
Hematocrito	NR	19,4%	19,4%
Gota gruesa	*Plasmodium vivax *(+++)	Negativo	NR
Prueba rápida VIH	NR	Negativo	NR

NR: no realizado

**Tabla 2:** Exámenes laboratoriales en muestra de líquido cefalorraquídeo.

Tipo de examen	Resultado
Leucocitos	2 leucocitos/mm³
Hematíes	10 x 12 x campo
Glucosa	78,3 mg/dL
Proteínas	28,5 mg/dL
Tinción Gram	No se observaron gérmenes
Tinción Bk	No se observaron BAAR
Tinta China	Negativo

BAAR: bacilo acido alcohol resistente

## DISCUSIÓN

La MC es causada mayormente por una infección grave de *Plasmodium falciparum*, la inflamación cumple un papel significativo en el proceso de daño neuronal. Los síntomas clínicos son heterogéneos y los resultados van desde la recuperación completa hasta variadas secuelas neurológicas y, a veces, la muerte ^7^ ^,^ ^8^. Clásicamente, la demostración de MC por *Plasmodium vivax* se atribuye a una infección mixta con los parásitos de *Plasmodium falciparum*. Por lo que es recomendable confirmar la monoinfección del *Plasmodium vivax* con exámenes confiables como la reacción en cadena de polimerasa (PCR); sin embargo, la respuesta al tratamiento específico contra la infección por *Plasmodium vivax* pudiera explicar la monoinfección ^1^ ^,^ ^15^. Se presenta un caso de malaria por *Plasmodium vivax* detectado con métodos convencionales, que se manifestó con síntomas neurológicos y que respondió a la terapia antipalúdica.

El paciente tuvo varios episodios de convulsiones, seguidos de confusión y escasa respuesta al estímulo, similar a otros pacientes con infección aguda que desarrollan encefalopatía difusa, coma progresivo rápido y/o convulsiones sin volver a la conciencia. En algunos casos también existen signos neurológicos focales ^9^. Sin embargo, la convulsión no es un criterio para el diagnóstico de MC, sino de malaria grave. Por lo que, al relacionar el trastorno del sensorio en un paciente febril procedente de una zona endémica de malaria, sin otra patología que lo explique, se consideró el diagnóstico de MC.

En una serie de casos en la India que se realizó entre el 2008 y 2010, trece niños fueron diagnosticados con MC por *Plasmodium vivax* confirmado por PCR. En once de ellos las manifestaciones neurológicas fueron múltiples convulsiones, y siete además tuvieron signos de afección de primera motoneurona, sin evidencia de hemorragia o papiledema ^10^. Sin embargo, en un reporte de caso del mismo país en 2016, un niño de 10 años presentó hemorragia multifocal con compromiso profundo del sensorio y secuelas neurológicas como el incremento del tono muscular y estado de conciencia mínimo ^2^.

La anemia y la disminución en el recuento de plaquetas son sugestivos de malaria severa, la presencia de leucopenia en el reporte nos evidencia una depresión de las tres series sanguíneas. No existen muchos reportes de casos en la cual se encuentren alteradas las tres series celulares sanguíneas cuya única causa sea la malaria ^11^. Dado que este patrón es inusual, se realizó una evaluación hematológica para tratar de encontrar una causa externa a la enfermedad, pero el reporte del especialista no sugiere ninguna causa no propia del cuadro descrito. 

En la gran mayoría de casos reportados, el diagnóstico inicial se efectuó por gota gruesa y extendido en lámina de sangre periférica con la observación directa del parásito, y la confirmación de la especie a través de técnicas moleculares (PCR) ^1^ ^,^ ^2^ ^,^ ^11^ ^-^ ^13^. La respuesta al tratamiento con artesunato y clindamicina fue óptima, cabe resaltar que el uso de artesunato es una constante en el tratamiento por malaria grave ^1^ ^,^ ^2^ ^,^ ^11^ ^-^ ^13^ ^) ^con variación de la segunda droga, pudiendo ser esta clindamicina ^2^, doxiciclina ^1^ ^,^ ^13^, lumefantrina ^12^ e, inclusive, quinina y primaquina, sin el uso de artesunato ^14^, todos estos esquemas han demostrado mejoría del paciente y negativización del parásito en la sangre.

Como limitación del reporte se debe mencionar que no fue posible realizar PCR para el diagnóstico de MC, debido a la limitada infraestructura médica en la zona. 

En este caso se identificó a *Plasmodium vivax* como responsable de MC en este paciente. Se debe considerar a este parásito en el diagnóstico presuntivo de casos con afectación neurológica en zonas tropicales endémicas y no pensar solo en *Plasmodium falciparum* como único causante de esta complicación.
